# 4-Methyl-*N*-(9-methyl-9-aza­bicyclo­[3.3.1]non-3-yl)benzamide

**DOI:** 10.1107/S1600536812017795

**Published:** 2012-05-19

**Authors:** Diya Lv, Yan Cao, Xin Dong, Ziyang Lou, Yifeng Chai

**Affiliations:** a Department of Medicinal Chemistry, School of Pharmacy, Second Military Medical University, Shanghai 200433, People’s Republic of China

## Abstract

The asymmetric unit of the title compound, C_17_H_24_N_2_O, contains three independent mol­ecules. In the crystal, mol­ecules are linked by weak N—H⋯O hydrogen bonds into chains parallel to the *c* axis.

## Related literature

For background to our work to design and synthesize a series of potent 5-HT_3_ receptor antagonists, see: Bermudez *et al.* (1990 ▶); Vernekar *et al.* (2010 ▶); Yang *et al.* (2010 ▶).
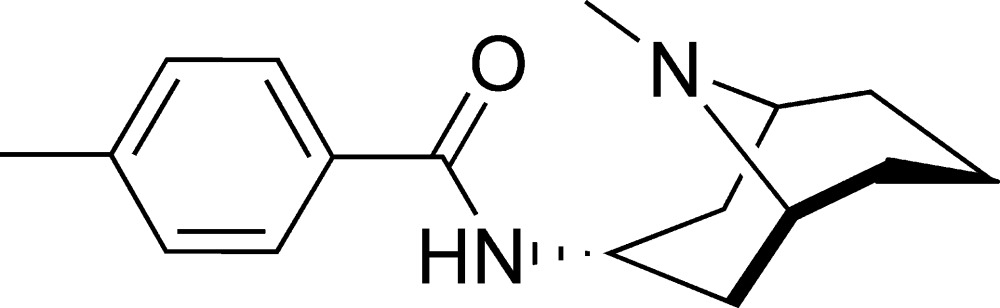


## Experimental

### 

#### Crystal data

C_17_H_24_N_2_O
*M* *_r_* = 272.38
Monoclinic, 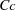

*a* = 38.68 (2) Å
*b* = 12.300 (7) Å
*c* = 9.975 (5) Å
β = 101.263 (7)°
*V* = 4654 (4) Å^3^
*Z* = 12
Mo *K*α radiation
μ = 0.07 mm^−1^
*T* = 293 K
0.25 × 0.15 × 0.10 mm

#### Data collection

Bruker SMART CCD area-detector diffractometer
Absorption correction: multi-scan (*SADABS*; Bruker, 1999 ▶) *T*_min_ = 0.982, *T*_max_ = 0.993
10320 measured reflections
6399 independent reflections
4035 reflections with *I* > 2σ(*I*)
*R*_int_ = 0.049

#### Refinement

*R*[*F* ^2^ > 2σ(*F* ^2^)] = 0.051
*wR*(*F* ^2^) = 0.151
*S* = 0.96
6399 reflections
556 parameters
5 restraints
H atoms treated by a mixture of independent and constrained refinement
Δρ_max_ = 0.20 e Å^−3^
Δρ_min_ = −0.23 e Å^−3^

### 

Data collection: *SMART* (Bruker, 1999 ▶); cell refinement: *SAINT* (Bruker, 1999 ▶); data reduction: *SAINT*; program(s) used to solve structure: *SHELXS97* (Sheldrick, 2008 ▶); program(s) used to refine structure: *SHELXL97* (Sheldrick, 2008 ▶); molecular graphics: *SHELXTL* (Sheldrick, 2008 ▶); software used to prepare material for publication: *SHELXL97*.

## Supplementary Material

Crystal structure: contains datablock(s) I, global. DOI: 10.1107/S1600536812017795/vm2165sup1.cif

Supplementary material file. DOI: 10.1107/S1600536812017795/vm2165Isup2.cdx

Structure factors: contains datablock(s) I. DOI: 10.1107/S1600536812017795/vm2165Isup3.hkl

Supplementary material file. DOI: 10.1107/S1600536812017795/vm2165Isup4.cml

Additional supplementary materials:  crystallographic information; 3D view; checkCIF report

## Figures and Tables

**Table 1:** Hydrogen-bond geometry (Å, °)

*D*—H⋯*A*	*D*—H	H⋯*A*	*D*⋯*A*	*D*—H⋯*A*
N2—H2′⋯O1^i^	0.85 (2)	2.10 (2)	2.935 (5)	171 (4)
N4—H4′⋯O2^ii^	0.86 (2)	2.05 (2)	2.894 (5)	166 (4)
N6—H6′⋯O3^iii^	0.86 (2)	2.30 (2)	3.164 (5)	174 (4)

Symmetry codes: (i) 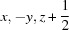; (ii) 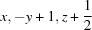; (iii) 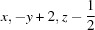.

**Fig. 1.**
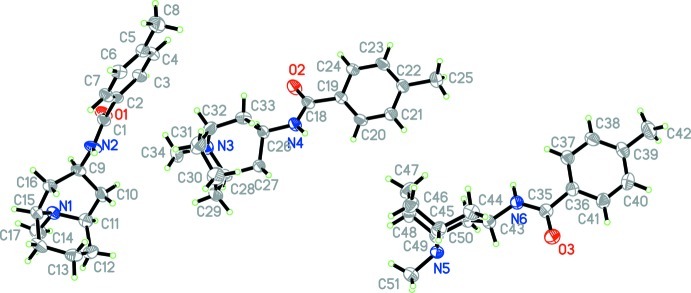
The molecular structure of the title compound with atom labels and 30% probability displacement ellipsoids for non-H atoms. The asymmetric unit contains three molecules.

**Fig. 2.**
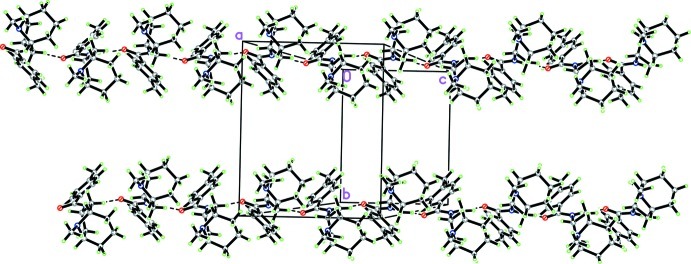
Intermolecular N2—H2···O1, N4—H4···O2 and N6—H6···O3 contacts forming a supramolecular chains along the c axis.

## supplementary crystallographic information

### Comment

Motion sickness is common in the population, especially with children. In our laboratory, we have designed and synthesized a novel series of potent 5-HT_3_ receptor antagonists (Bermudez *et al.*, 1990; Vernekar *et al.*, 2010; Yang *et al.*, 2010). Furthermore, we find that the candidate has the anti-motion sickness effect. Here, we report the crystal structure of the title compound.

As shown in Fig.1, the asymmetric unit of the title compound contains three molecules. H-bonding interactions do play a decisive role in the crystal packing arrangement (Fig. 2). The molecules are linked by weak intermolecular N2—H2···O1, N4—H4···O2 and N6—H6···O3 hydrogen bonds (Table 1) linking the molecules into chains parallel to the *c* axis.

### Experimental

A solution of 9-methyl-9-azabicyclo[3.3.1]nonan-3-one (0.03 mol) in ethanol (150 ml) was added dropwise to a solution of sodium acetate (0.06 mol) and hydroxylamine hydrochloride (0.06 mol) in ethanol (200 ml) under stirring, and refluxed for 4 hr to get crude products, which was recrystallized from ethyl acetate to give pure 9-methyl-9-azabicyclo[3.3.1]nonan-3-one (3.8 g, yield 75.2%). To a solution of LiAlH_4_ (0.066 mol) in anhydrous tetrahydrofuran (THF, 50 ml) a solution of concentrated sulfuric acid (3.0 ml) and THF (12 ml) was added dropwise at -10°C and stirred for 1 hr. To thisabove reaction mixture, a solution of 9-methyl-9-azbicyclo[3.3.1]non-3-ylamine (0.022 mol) in anhydrous THF (70 ml) was added dropwise at 30-35°C and stirred. After reaction, the reaction mixture was evaporated at vacuum to remove solvent to give 3-amine-9-methyl-9-azabicyclo[3.3.1]nonane (2.9 g, yield 78.3%). To a solution of 3-amine-9-methyl-9-azabicyclo[3.3.1]nonane (0.013 mol) and triethylamine (0.013 mol) in dichloromethane (20 ml), a mixture of 4-methylbenzoyl chloride (0.016 mol) and dichloromethane (5 ml) was added dropwise and stirred to obtain crude product, which was recrystallized from ethyl acetate to give pure 4-methyl-*N*-(9-methyl-9-azabicyclo[3.3.1]non-3-yl)-benzenecarboxamide (2.9 g, yield 81%).

### Refinement

All hydrogen atoms were placed in calculated positions using a riding model, with d(C—H) = 0.93 Å for aromatic, 0.97 Å for CH_2_ and 0.96 Å for CH_3_ groups and d(N—H) = 0.86 Å for NH, and with *U*_iso_(H) = 1.2U_eq_(C) and *U*_iso_(H) = 1.5U_eq_(N).

### Crystal data

**Table:**

C_17_H_24_N_2_O	*F*(000) = 1776
*M**_r_* = 272.38	*D*_x_ = 1.166 Mg m^−^^3^
Monoclinic, *C**c*	Mo *K*α radiation, λ = 0.71073 Å
Hall symbol: C -2yc	Cell parameters from 2865 reflections
*a* = 38.68 (2) Å	θ = 2.3–24.1°
*b* = 12.300 (7) Å	µ = 0.07 mm^−^^1^
*c* = 9.975 (5) Å	*T* = 293 K
β = 101.263 (7)°	Prism, colorless
*V* = 4654 (4) Å^3^	0.25 × 0.15 × 0.10 mm
*Z* = 12	

### Data collection

**Table:**

Bruker SMART CCD area-detector diffractometer	6399 independent reflections
Radiation source: fine-focus sealed tube	4035 reflections with *I* > 2σ(*I*)
Graphite monochromator	*R*_int_ = 0.049
phi and ω scans	θ_max_ = 26.0°, θ_min_ = 1.7°
Absorption correction: multi-scan (*SADABS*; Bruker, 1999)	*h* = −36→47
*T*_min_ = 0.982, *T*_max_ = 0.993	*k* = −15→15
10320 measured reflections	*l* = −12→11

### Refinement

**Table:**

Refinement on *F*^2^	Secondary atom site location: difference Fourier map
Least-squares matrix: full	Hydrogen site location: inferred from neighbouring sites
*R*[*F*^2^ > 2σ(*F*^2^)] = 0.051	H atoms treated by a mixture of independent and constrained refinement
*wR*(*F*^2^) = 0.151	*w* = 1/[σ^2^(*F*_o_^2^) + (0.0788*P*)^2^] where *P* = (*F*_o_^2^ + 2*F*_c_^2^)/3
*S* = 0.96	(Δ/σ)_max_ = 0.001
6399 reflections	Δρ_max_ = 0.20 e Å^−^^3^
556 parameters	Δρ_min_ = −0.23 e Å^−^^3^
5 restraints	Absolute structure: Flack, H. D. (1983). *Acta Cryst.* A39, 876–881.
Primary atom site location: structure-invariant direct methods	Flack parameter: −3.4 (17)

### Special details

**Table:**

Geometry. All e.s.d.'s (except the e.s.d. in the dihedral angle between two l.s. planes) are estimated using the full covariance matrix. The cell e.s.d.'s are taken into account individually in the estimation of e.s.d.'s in distances, angles and torsion angles; correlations between e.s.d.'s in cell parameters are only used when they are defined by crystal symmetry. An approximate (isotropic) treatment of cell e.s.d.'s is used for estimating e.s.d.'s involving l.s. planes.
Refinement. Refinement of *F*^2^ against ALL reflections. The weighted *R*-factor *wR* and goodness of fit *S* are based on *F*^2^, conventional *R*-factors *R* are based on *F*, with *F* set to zero for negative *F*^2^. The threshold expression of *F*^2^ > σ(*F*^2^) is used only for calculating *R*-factors(gt) *etc*. and is not relevant to the choice of reflections for refinement. *R*-factors based on *F*^2^ are statistically about twice as large as those based on *F*, and *R*- factors based on ALL data will be even larger.

### Fractional atomic coordinates and isotropic or equivalent isotropic displacement parameters (Å^2^)

**Table:**

	*x*	*y*	*z*	*U*_iso_*/*U*_eq_	
N1	0.58077 (8)	0.1326 (3)	0.3192 (3)	0.0543 (8)	
N2	0.48226 (9)	0.0124 (3)	0.2549 (3)	0.0609 (9)	
H2'	0.4764 (11)	−0.005 (3)	0.330 (3)	0.073*	
O1	0.46853 (7)	0.0334 (3)	0.0285 (3)	0.0705 (8)	
C1	0.46056 (10)	0.0018 (3)	0.1364 (4)	0.0528 (10)	
C2	0.42609 (10)	−0.0505 (3)	0.1370 (4)	0.0488 (9)	
C3	0.39749 (11)	−0.0253 (3)	0.0334 (4)	0.0589 (11)	
H3	0.4003	0.0242	−0.0343	0.071*	
C4	0.36548 (11)	−0.0725 (4)	0.0305 (5)	0.0670 (12)	
H4	0.3467	−0.0528	−0.0383	0.080*	
C5	0.35993 (10)	−0.1489 (3)	0.1261 (5)	0.0627 (12)	
C6	0.38855 (11)	−0.1749 (3)	0.2235 (4)	0.0619 (11)	
H6	0.3862	−0.2279	0.2876	0.074*	
C7	0.42059 (10)	−0.1264 (3)	0.2308 (4)	0.0558 (10)	
H7	0.4391	−0.1454	0.3012	0.067*	
C8	0.32499 (12)	−0.2024 (4)	0.1200 (6)	0.0900 (16)	
H8A	0.3085	−0.1503	0.1421	0.135*	
H8B	0.3275	−0.2612	0.1843	0.135*	
H8C	0.3166	−0.2300	0.0295	0.135*	
C9	0.51759 (10)	0.0560 (3)	0.2706 (4)	0.0531 (10)	
H9	0.5255	0.0471	0.1837	0.064*	
C10	0.51907 (11)	0.1753 (3)	0.3063 (4)	0.0626 (11)	
H10A	0.5076	0.1868	0.3832	0.075*	
H10B	0.5062	0.2161	0.2293	0.075*	
C11	0.55702 (11)	0.2186 (3)	0.3427 (4)	0.0613 (11)	
H11	0.5592	0.2782	0.2797	0.074*	
C12	0.56556 (13)	0.2638 (3)	0.4878 (5)	0.0736 (13)	
H12A	0.5885	0.2986	0.5031	0.088*	
H12B	0.5482	0.3185	0.4986	0.088*	
C13	0.56561 (13)	0.1746 (4)	0.5929 (4)	0.0740 (13)	
H13A	0.5746	0.2032	0.6837	0.089*	
H13B	0.5417	0.1496	0.5898	0.089*	
C14	0.58816 (11)	0.0809 (4)	0.5652 (5)	0.0715 (13)	
H14A	0.5849	0.0204	0.6238	0.086*	
H14B	0.6128	0.1023	0.5874	0.086*	
C15	0.57904 (10)	0.0439 (3)	0.4144 (4)	0.0566 (10)	
H15	0.5963	−0.0112	0.4007	0.068*	
C16	0.54243 (10)	−0.0080 (3)	0.3798 (4)	0.0531 (10)	
H16A	0.5446	−0.0817	0.3478	0.064*	
H16B	0.5325	−0.0116	0.4619	0.064*	
C17	0.61658 (12)	0.1692 (4)	0.3199 (5)	0.0852 (15)	
H17A	0.6275	0.1913	0.4106	0.128*	
H17B	0.6160	0.2297	0.2586	0.128*	
H17C	0.6299	0.1109	0.2909	0.128*	
N3	0.40602 (8)	0.3625 (3)	0.0728 (3)	0.0606 (9)	
N4	0.30958 (8)	0.4950 (3)	0.0227 (3)	0.0552 (8)	
H4'	0.3019 (10)	0.506 (3)	0.097 (3)	0.066*	
O2	0.29556 (7)	0.4833 (3)	−0.2040 (3)	0.0750 (9)	
C18	0.28783 (9)	0.5105 (3)	−0.0962 (4)	0.0521 (10)	
C19	0.25307 (9)	0.5615 (3)	−0.0922 (4)	0.0477 (9)	
C20	0.24716 (10)	0.6300 (3)	0.0099 (4)	0.0552 (10)	
H20	0.2655	0.6458	0.0823	0.066*	
C21	0.21491 (11)	0.6750 (3)	0.0068 (4)	0.0605 (11)	
H21	0.2120	0.7227	0.0761	0.073*	
C22	0.18671 (10)	0.6526 (3)	−0.0946 (4)	0.0565 (10)	
C23	0.19269 (11)	0.5849 (3)	−0.1974 (5)	0.0664 (12)	
H23	0.1741	0.5693	−0.2693	0.080*	
C24	0.22492 (10)	0.5399 (3)	−0.1972 (4)	0.0579 (11)	
H24	0.2280	0.4943	−0.2684	0.069*	
C25	0.15105 (12)	0.7020 (4)	−0.0963 (6)	0.0857 (15)	
H25A	0.1509	0.7360	−0.0098	0.129*	
H25B	0.1334	0.6462	−0.1125	0.129*	
H25C	0.1461	0.7554	−0.1677	0.129*	
C26	0.34431 (9)	0.4478 (3)	0.0351 (4)	0.0491 (9)	
H26	0.3517	0.4550	−0.0531	0.059*	
C27	0.37066 (10)	0.5075 (3)	0.1421 (4)	0.0515 (9)	
H27A	0.3617	0.5114	0.2262	0.062*	
H27B	0.3733	0.5813	0.1112	0.062*	
C28	0.40679 (10)	0.4522 (3)	0.1711 (4)	0.0564 (10)	
H28	0.4243	0.5053	0.1541	0.068*	
C29	0.41717 (12)	0.4145 (4)	0.3186 (4)	0.0793 (15)	
H29A	0.4415	0.3904	0.3358	0.095*	
H29B	0.4155	0.4753	0.3789	0.095*	
C30	0.39411 (15)	0.3229 (4)	0.3508 (5)	0.0883 (16)	
H30A	0.3708	0.3507	0.3535	0.106*	
H30B	0.4040	0.2931	0.4402	0.106*	
C31	0.39119 (16)	0.2330 (4)	0.2435 (5)	0.0894 (16)	
H31A	0.4135	0.1952	0.2533	0.107*	
H31B	0.3734	0.1808	0.2577	0.107*	
C32	0.38135 (12)	0.2796 (3)	0.1010 (4)	0.0660 (12)	
H32	0.3818	0.2199	0.0363	0.079*	
C33	0.34430 (11)	0.3284 (3)	0.0711 (4)	0.0641 (11)	
H33A	0.3300	0.2891	−0.0042	0.077*	
H33B	0.3336	0.3192	0.1507	0.077*	
C34	0.44072 (13)	0.3213 (5)	0.0673 (5)	0.1015 (19)	
H34A	0.4530	0.3038	0.1579	0.152*	
H34B	0.4537	0.3756	0.0287	0.152*	
H34C	0.4385	0.2572	0.0114	0.152*	
N5	0.24691 (9)	0.9123 (3)	0.6198 (3)	0.0589 (9)	
N6	0.14766 (9)	1.0004 (3)	0.4521 (3)	0.0581 (9)	
H6'	0.1420 (11)	0.983 (3)	0.367 (2)	0.070*	
O3	0.13264 (8)	1.0699 (2)	0.6410 (3)	0.0708 (8)	
C35	0.12497 (11)	1.0463 (3)	0.5175 (4)	0.0548 (10)	
C36	0.08923 (10)	1.0718 (3)	0.4366 (4)	0.0527 (9)	
C37	0.07519 (11)	1.0213 (3)	0.3145 (4)	0.0597 (11)	
H37	0.0882	0.9685	0.2792	0.072*	
C38	0.04217 (11)	1.0486 (3)	0.2451 (5)	0.0671 (12)	
H38	0.0330	1.0124	0.1641	0.081*	
C39	0.02218 (11)	1.1268 (3)	0.2901 (5)	0.0662 (12)	
C40	0.03636 (12)	1.1764 (4)	0.4127 (5)	0.0719 (13)	
H40	0.0233	1.2290	0.4475	0.086*	
C41	0.06940 (11)	1.1500 (3)	0.4847 (5)	0.0660 (12)	
H41	0.0784	1.1854	0.5666	0.079*	
C42	−0.01294 (13)	1.1604 (5)	0.2088 (6)	0.1033 (18)	
H42A	−0.0133	1.2378	0.1961	0.155*	
H42B	−0.0312	1.1399	0.2568	0.155*	
H42C	−0.0169	1.1251	0.1213	0.155*	
C43	0.18360 (10)	0.9727 (3)	0.5173 (4)	0.0550 (10)	
H43	0.1904	1.0192	0.5979	0.066*	
C44	0.18626 (11)	0.8552 (3)	0.5649 (5)	0.0689 (12)	
H44A	0.1755	0.8088	0.4896	0.083*	
H44B	0.1731	0.8465	0.6377	0.083*	
C45	0.22420 (11)	0.8184 (3)	0.6162 (4)	0.0586 (11)	
H45	0.2262	0.7917	0.7100	0.070*	
C46	0.23481 (14)	0.7258 (3)	0.5295 (5)	0.0803 (14)	
H46A	0.2575	0.6972	0.5739	0.096*	
H46B	0.2177	0.6675	0.5232	0.096*	
C47	0.23692 (15)	0.7642 (4)	0.3866 (5)	0.0825 (15)	
H47A	0.2477	0.7079	0.3406	0.099*	
H47B	0.2133	0.7763	0.3348	0.099*	
C48	0.25764 (13)	0.8662 (4)	0.3886 (5)	0.0762 (13)	
H48A	0.2549	0.8945	0.2964	0.091*	
H48B	0.2824	0.8505	0.4213	0.091*	
C49	0.24582 (11)	0.9519 (3)	0.4801 (4)	0.0606 (11)	
H49	0.2621	1.0134	0.4857	0.073*	
C50	0.20874 (11)	0.9945 (3)	0.4227 (4)	0.0607 (11)	
H50A	0.2099	1.0722	0.4075	0.073*	
H50B	0.1999	0.9601	0.3352	0.073*	
C51	0.28263 (12)	0.8941 (4)	0.6964 (5)	0.0897 (16)	
H51A	0.2938	0.8385	0.6523	0.134*	
H51B	0.2817	0.8716	0.7878	0.134*	
H51C	0.2959	0.9603	0.6995	0.134*	

### Atomic displacement parameters (Å^2^)

**Table:**

	*U*^11^	*U*^22^	*U*^33^	*U*^12^	*U*^13^	*U*^23^
N1	0.0452 (19)	0.0620 (19)	0.055 (2)	−0.0041 (16)	0.0069 (15)	0.0034 (16)
N2	0.050 (2)	0.103 (3)	0.0281 (18)	−0.0168 (18)	0.0040 (15)	−0.0049 (18)
O1	0.0581 (18)	0.121 (2)	0.0312 (16)	−0.0092 (16)	0.0058 (13)	0.0028 (15)
C1	0.044 (2)	0.074 (3)	0.038 (3)	0.0028 (19)	0.0038 (18)	−0.0093 (19)
C2	0.045 (2)	0.063 (2)	0.037 (2)	0.0000 (18)	0.0044 (17)	−0.0100 (17)
C3	0.054 (3)	0.068 (2)	0.049 (2)	−0.003 (2)	−0.003 (2)	−0.0019 (19)
C4	0.051 (3)	0.073 (3)	0.068 (3)	0.004 (2)	−0.011 (2)	−0.003 (2)
C5	0.046 (3)	0.058 (2)	0.082 (3)	−0.005 (2)	0.007 (2)	−0.015 (2)
C6	0.062 (3)	0.069 (3)	0.054 (3)	−0.011 (2)	0.011 (2)	−0.003 (2)
C7	0.047 (2)	0.078 (3)	0.040 (2)	0.001 (2)	0.0022 (17)	0.000 (2)
C8	0.061 (3)	0.080 (3)	0.126 (5)	−0.009 (2)	0.010 (3)	−0.005 (3)
C9	0.047 (2)	0.080 (3)	0.032 (2)	−0.009 (2)	0.0050 (16)	−0.0006 (18)
C10	0.058 (3)	0.070 (3)	0.056 (3)	0.016 (2)	0.0031 (19)	0.010 (2)
C11	0.066 (3)	0.057 (2)	0.059 (3)	−0.004 (2)	0.010 (2)	0.010 (2)
C12	0.086 (3)	0.064 (3)	0.070 (3)	−0.005 (2)	0.012 (2)	−0.013 (2)
C13	0.082 (3)	0.090 (3)	0.046 (3)	−0.009 (3)	0.003 (2)	−0.010 (2)
C14	0.054 (3)	0.087 (3)	0.064 (3)	−0.010 (2)	−0.010 (2)	0.015 (2)
C15	0.047 (2)	0.063 (2)	0.056 (3)	0.0041 (19)	0.0013 (18)	0.0006 (19)
C16	0.059 (3)	0.059 (2)	0.041 (2)	−0.0020 (18)	0.0070 (17)	−0.0030 (17)
C17	0.063 (3)	0.097 (3)	0.095 (4)	−0.017 (3)	0.015 (3)	0.003 (3)
N3	0.056 (2)	0.082 (2)	0.044 (2)	0.0203 (19)	0.0089 (15)	−0.0063 (17)
N4	0.048 (2)	0.083 (2)	0.0340 (19)	0.0071 (16)	0.0073 (15)	0.0019 (16)
O2	0.0605 (19)	0.135 (3)	0.0301 (16)	0.0110 (17)	0.0112 (13)	−0.0036 (16)
C18	0.044 (2)	0.068 (2)	0.043 (2)	−0.0034 (18)	0.0063 (18)	0.0051 (19)
C19	0.044 (2)	0.058 (2)	0.041 (2)	−0.0037 (17)	0.0076 (17)	0.0080 (18)
C20	0.044 (2)	0.073 (3)	0.045 (2)	−0.005 (2)	−0.0009 (17)	−0.004 (2)
C21	0.056 (3)	0.071 (2)	0.054 (3)	0.002 (2)	0.010 (2)	−0.008 (2)
C22	0.046 (2)	0.053 (2)	0.069 (3)	−0.0010 (18)	0.007 (2)	0.005 (2)
C23	0.052 (3)	0.070 (3)	0.067 (3)	0.002 (2)	−0.014 (2)	0.000 (2)
C24	0.054 (3)	0.067 (2)	0.046 (2)	0.006 (2)	−0.0058 (19)	−0.0047 (19)
C25	0.063 (3)	0.082 (3)	0.109 (4)	0.012 (3)	0.009 (3)	−0.009 (3)
C26	0.043 (2)	0.064 (2)	0.041 (2)	0.0059 (18)	0.0099 (16)	0.0014 (18)
C27	0.048 (2)	0.058 (2)	0.048 (2)	0.0019 (18)	0.0087 (17)	−0.0035 (18)
C28	0.048 (2)	0.076 (3)	0.042 (2)	0.002 (2)	0.0009 (17)	−0.004 (2)
C29	0.070 (3)	0.108 (4)	0.049 (3)	0.025 (3)	−0.015 (2)	−0.016 (3)
C30	0.116 (4)	0.099 (4)	0.048 (3)	0.021 (3)	0.010 (3)	0.014 (3)
C31	0.126 (5)	0.076 (3)	0.064 (3)	0.028 (3)	0.013 (3)	0.011 (3)
C32	0.086 (3)	0.059 (2)	0.052 (3)	0.013 (2)	0.010 (2)	−0.0075 (19)
C33	0.070 (3)	0.069 (2)	0.051 (2)	−0.013 (2)	0.008 (2)	−0.005 (2)
C34	0.076 (4)	0.146 (5)	0.078 (4)	0.051 (4)	0.005 (3)	−0.015 (3)
N5	0.057 (2)	0.072 (2)	0.0435 (19)	0.0100 (17)	−0.0014 (15)	−0.0080 (17)
N6	0.054 (2)	0.082 (2)	0.0374 (19)	0.0109 (18)	0.0061 (16)	−0.0024 (18)
O3	0.0744 (19)	0.100 (2)	0.0400 (18)	0.0044 (16)	0.0157 (14)	−0.0029 (15)
C35	0.057 (3)	0.060 (2)	0.049 (3)	−0.002 (2)	0.014 (2)	0.0064 (19)
C36	0.052 (2)	0.055 (2)	0.054 (3)	−0.0003 (19)	0.0161 (18)	0.0068 (19)
C37	0.057 (3)	0.058 (2)	0.065 (3)	0.000 (2)	0.012 (2)	0.003 (2)
C38	0.055 (3)	0.066 (2)	0.076 (3)	−0.008 (2)	0.003 (2)	0.000 (2)
C39	0.049 (3)	0.069 (3)	0.082 (3)	−0.001 (2)	0.014 (2)	0.012 (3)
C40	0.073 (3)	0.069 (3)	0.081 (4)	0.012 (2)	0.034 (3)	0.001 (2)
C41	0.064 (3)	0.072 (3)	0.064 (3)	0.004 (2)	0.020 (2)	−0.001 (2)
C42	0.061 (3)	0.114 (4)	0.131 (5)	0.012 (3)	0.008 (3)	0.004 (4)
C43	0.054 (3)	0.064 (2)	0.048 (2)	0.0013 (19)	0.0100 (18)	0.0045 (18)
C44	0.070 (3)	0.068 (3)	0.068 (3)	−0.007 (2)	0.012 (2)	0.009 (2)
C45	0.075 (3)	0.067 (2)	0.034 (2)	0.010 (2)	0.0103 (19)	0.0074 (18)
C46	0.095 (4)	0.058 (3)	0.084 (4)	0.009 (2)	0.008 (3)	−0.007 (2)
C47	0.115 (4)	0.081 (3)	0.051 (3)	0.025 (3)	0.016 (3)	−0.014 (2)
C48	0.077 (3)	0.097 (3)	0.059 (3)	0.025 (3)	0.025 (2)	0.008 (2)
C49	0.057 (3)	0.063 (2)	0.061 (3)	0.001 (2)	0.009 (2)	0.003 (2)
C50	0.060 (3)	0.061 (2)	0.061 (3)	0.005 (2)	0.012 (2)	0.007 (2)
C51	0.073 (3)	0.105 (4)	0.078 (4)	0.021 (3)	−0.015 (3)	−0.017 (3)

### Geometric parameters (Å, º)

**Table:**

N1—C11	1.449 (5)	C26—C27	1.515 (5)
N1—C17	1.456 (5)	C26—H26	0.9800
N1—C15	1.456 (5)	C27—C28	1.530 (5)
N2—C1	1.315 (5)	C27—H27A	0.9700
N2—C9	1.448 (5)	C27—H27B	0.9700
N2—H2'	0.846 (19)	C28—C29	1.520 (6)
O1—C1	1.239 (5)	C28—H28	0.9800
C1—C2	1.482 (5)	C29—C30	1.511 (7)
C2—C7	1.367 (5)	C29—H29A	0.9700
C2—C3	1.394 (5)	C29—H29B	0.9700
C3—C4	1.363 (6)	C30—C31	1.528 (7)
C3—H3	0.9300	C30—H30A	0.9700
C4—C5	1.385 (6)	C30—H30B	0.9700
C4—H4	0.9300	C31—C32	1.510 (6)
C5—C6	1.361 (5)	C31—H31A	0.9700
C5—C8	1.493 (6)	C31—H31B	0.9700
C6—C7	1.365 (5)	C32—C33	1.528 (6)
C6—H6	0.9300	C32—H32	0.9800
C7—H7	0.9300	C33—H33A	0.9700
C8—H8A	0.9600	C33—H33B	0.9700
C8—H8B	0.9600	C34—H34A	0.9600
C8—H8C	0.9600	C34—H34B	0.9600
C9—C10	1.508 (5)	C34—H34C	0.9600
C9—C16	1.523 (5)	N5—C45	1.447 (5)
C9—H9	0.9800	N5—C51	1.459 (5)
C10—C11	1.537 (6)	N5—C49	1.469 (5)
C10—H10A	0.9700	N6—C35	1.319 (5)
C10—H10B	0.9700	N6—C43	1.456 (5)
C11—C12	1.525 (6)	N6—H6'	0.864 (19)
C11—H11	0.9800	O3—C35	1.243 (5)
C12—C13	1.518 (6)	C35—C36	1.492 (5)
C12—H12A	0.9700	C36—C41	1.374 (5)
C12—H12B	0.9700	C36—C37	1.381 (6)
C13—C14	1.503 (6)	C37—C38	1.370 (5)
C13—H13A	0.9700	C37—H37	0.9300
C13—H13B	0.9700	C38—C39	1.364 (6)
C14—C15	1.545 (6)	C38—H38	0.9300
C14—H14A	0.9700	C39—C40	1.381 (6)
C14—H14B	0.9700	C39—C42	1.498 (6)
C15—C16	1.530 (5)	C40—C41	1.377 (6)
C15—H15	0.9800	C40—H40	0.9300
C16—H16A	0.9700	C41—H41	0.9300
C16—H16B	0.9700	C42—H42A	0.9600
C17—H17A	0.9600	C42—H42B	0.9600
C17—H17B	0.9600	C42—H42C	0.9600
C17—H17C	0.9600	C43—C50	1.506 (6)
N3—C34	1.446 (5)	C43—C44	1.518 (6)
N3—C32	1.461 (5)	C43—H43	0.9800
N3—C28	1.472 (5)	C44—C45	1.526 (6)
N4—C18	1.327 (5)	C44—H44A	0.9700
N4—C26	1.447 (5)	C44—H44B	0.9700
N4—H4'	0.859 (19)	C45—C46	1.534 (6)
O2—C18	1.218 (5)	C45—H45	0.9800
C18—C19	1.491 (5)	C46—C47	1.520 (7)
C19—C20	1.375 (5)	C46—H46A	0.9700
C19—C24	1.382 (5)	C46—H46B	0.9700
C20—C21	1.360 (5)	C47—C48	1.487 (7)
C20—H20	0.9300	C47—H47A	0.9700
C21—C22	1.362 (5)	C47—H47B	0.9700
C21—H21	0.9300	C48—C49	1.521 (6)
C22—C23	1.375 (6)	C48—H48A	0.9700
C22—C25	1.504 (6)	C48—H48B	0.9700
C23—C24	1.364 (6)	C49—C50	1.529 (5)
C23—H23	0.9300	C49—H49	0.9800
C24—H24	0.9300	C50—H50A	0.9700
C25—H25A	0.9600	C50—H50B	0.9700
C25—H25B	0.9600	C51—H51A	0.9600
C25—H25C	0.9600	C51—H51B	0.9600
C26—C33	1.512 (5)	C51—H51C	0.9600
			
C11—N1—C17	114.0 (3)	H27A—C27—H27B	107.9
C11—N1—C15	109.3 (3)	N3—C28—C29	112.5 (3)
C17—N1—C15	113.2 (3)	N3—C28—C27	108.0 (3)
C1—N2—C9	123.8 (3)	C29—C28—C27	112.2 (4)
C1—N2—H2'	122 (3)	N3—C28—H28	108.0
C9—N2—H2'	114 (3)	C29—C28—H28	108.0
O1—C1—N2	121.8 (4)	C27—C28—H28	108.0
O1—C1—C2	121.1 (3)	C30—C29—C28	112.2 (4)
N2—C1—C2	117.1 (4)	C30—C29—H29A	109.2
C7—C2—C3	116.9 (4)	C28—C29—H29A	109.2
C7—C2—C1	124.1 (3)	C30—C29—H29B	109.2
C3—C2—C1	119.0 (4)	C28—C29—H29B	109.2
C4—C3—C2	120.5 (4)	H29A—C29—H29B	107.9
C4—C3—H3	119.8	C29—C30—C31	110.9 (4)
C2—C3—H3	119.8	C29—C30—H30A	109.5
C3—C4—C5	122.5 (4)	C31—C30—H30A	109.5
C3—C4—H4	118.8	C29—C30—H30B	109.5
C5—C4—H4	118.8	C31—C30—H30B	109.5
C6—C5—C4	115.9 (4)	H30A—C30—H30B	108.1
C6—C5—C8	121.9 (4)	C32—C31—C30	110.8 (4)
C4—C5—C8	122.1 (4)	C32—C31—H31A	109.5
C5—C6—C7	122.6 (4)	C30—C31—H31A	109.5
C5—C6—H6	118.7	C32—C31—H31B	109.5
C7—C6—H6	118.7	C30—C31—H31B	109.5
C6—C7—C2	121.6 (3)	H31A—C31—H31B	108.1
C6—C7—H7	119.2	N3—C32—C31	113.1 (4)
C2—C7—H7	119.2	N3—C32—C33	108.2 (3)
C5—C8—H8A	109.5	C31—C32—C33	112.8 (4)
C5—C8—H8B	109.5	N3—C32—H32	107.5
H8A—C8—H8B	109.5	C31—C32—H32	107.5
C5—C8—H8C	109.5	C33—C32—H32	107.5
H8A—C8—H8C	109.5	C26—C33—C32	112.6 (3)
H8B—C8—H8C	109.5	C26—C33—H33A	109.1
N2—C9—C10	112.2 (3)	C32—C33—H33A	109.1
N2—C9—C16	109.6 (3)	C26—C33—H33B	109.1
C10—C9—C16	110.1 (3)	C32—C33—H33B	109.1
N2—C9—H9	108.2	H33A—C33—H33B	107.8
C10—C9—H9	108.2	N3—C34—H34A	109.5
C16—C9—H9	108.2	N3—C34—H34B	109.5
C9—C10—C11	112.5 (3)	H34A—C34—H34B	109.5
C9—C10—H10A	109.1	N3—C34—H34C	109.5
C11—C10—H10A	109.1	H34A—C34—H34C	109.5
C9—C10—H10B	109.1	H34B—C34—H34C	109.5
C11—C10—H10B	109.1	C45—N5—C51	113.5 (3)
H10A—C10—H10B	107.8	C45—N5—C49	109.6 (3)
N1—C11—C12	113.2 (3)	C51—N5—C49	113.3 (4)
N1—C11—C10	108.4 (3)	C35—N6—C43	123.3 (3)
C12—C11—C10	111.7 (4)	C35—N6—H6'	122 (3)
N1—C11—H11	107.8	C43—N6—H6'	115 (3)
C12—C11—H11	107.8	O3—C35—N6	122.5 (4)
C10—C11—H11	107.8	O3—C35—C36	120.2 (4)
C13—C12—C11	111.2 (3)	N6—C35—C36	117.3 (4)
C13—C12—H12A	109.4	C41—C36—C37	118.3 (4)
C11—C12—H12A	109.4	C41—C36—C35	118.7 (4)
C13—C12—H12B	109.4	C37—C36—C35	123.1 (4)
C11—C12—H12B	109.4	C38—C37—C36	120.1 (4)
H12A—C12—H12B	108.0	C38—C37—H37	119.9
C14—C13—C12	110.5 (4)	C36—C37—H37	119.9
C14—C13—H13A	109.6	C39—C38—C37	122.6 (4)
C12—C13—H13A	109.6	C39—C38—H38	118.7
C14—C13—H13B	109.6	C37—C38—H38	118.7
C12—C13—H13B	109.6	C38—C39—C40	116.8 (4)
H13A—C13—H13B	108.1	C38—C39—C42	121.9 (5)
C13—C14—C15	112.0 (3)	C40—C39—C42	121.3 (5)
C13—C14—H14A	109.2	C41—C40—C39	121.7 (4)
C15—C14—H14A	109.2	C41—C40—H40	119.1
C13—C14—H14B	109.2	C39—C40—H40	119.1
C15—C14—H14B	109.2	C36—C41—C40	120.5 (4)
H14A—C14—H14B	107.9	C36—C41—H41	119.8
N1—C15—C16	108.9 (3)	C40—C41—H41	119.8
N1—C15—C14	112.5 (3)	C39—C42—H42A	109.5
C16—C15—C14	111.6 (4)	C39—C42—H42B	109.5
N1—C15—H15	107.9	H42A—C42—H42B	109.5
C16—C15—H15	107.9	C39—C42—H42C	109.5
C14—C15—H15	107.9	H42A—C42—H42C	109.5
C9—C16—C15	111.9 (3)	H42B—C42—H42C	109.5
C9—C16—H16A	109.2	N6—C43—C50	110.8 (3)
C15—C16—H16A	109.2	N6—C43—C44	111.3 (3)
C9—C16—H16B	109.2	C50—C43—C44	110.7 (4)
C15—C16—H16B	109.2	N6—C43—H43	108.0
H16A—C16—H16B	107.9	C50—C43—H43	108.0
N1—C17—H17A	109.5	C44—C43—H43	108.0
N1—C17—H17B	109.5	C43—C44—C45	112.9 (3)
H17A—C17—H17B	109.5	C43—C44—H44A	109.0
N1—C17—H17C	109.5	C45—C44—H44A	109.0
H17A—C17—H17C	109.5	C43—C44—H44B	109.0
H17B—C17—H17C	109.5	C45—C44—H44B	109.0
C34—N3—C32	114.1 (4)	H44A—C44—H44B	107.8
C34—N3—C28	113.0 (3)	N5—C45—C44	108.1 (3)
C32—N3—C28	108.9 (3)	N5—C45—C46	112.2 (4)
C18—N4—C26	123.4 (3)	C44—C45—C46	112.1 (3)
C18—N4—H4'	119 (3)	N5—C45—H45	108.1
C26—N4—H4'	117 (3)	C44—C45—H45	108.1
O2—C18—N4	121.8 (4)	C46—C45—H45	108.1
O2—C18—C19	121.2 (3)	C47—C46—C45	111.6 (3)
N4—C18—C19	117.0 (4)	C47—C46—H46A	109.3
C20—C19—C24	117.3 (3)	C45—C46—H46A	109.3
C20—C19—C18	124.0 (3)	C47—C46—H46B	109.3
C24—C19—C18	118.7 (4)	C45—C46—H46B	109.3
C21—C20—C19	121.1 (3)	H46A—C46—H46B	108.0
C21—C20—H20	119.4	C48—C47—C46	112.1 (4)
C19—C20—H20	119.4	C48—C47—H47A	109.2
C20—C21—C22	122.2 (4)	C46—C47—H47A	109.2
C20—C21—H21	118.9	C48—C47—H47B	109.2
C22—C21—H21	118.9	C46—C47—H47B	109.2
C21—C22—C23	116.7 (4)	H47A—C47—H47B	107.9
C21—C22—C25	121.8 (4)	C47—C48—C49	111.6 (4)
C23—C22—C25	121.6 (4)	C47—C48—H48A	109.3
C24—C23—C22	122.1 (4)	C49—C48—H48A	109.3
C24—C23—H23	118.9	C47—C48—H48B	109.3
C22—C23—H23	118.9	C49—C48—H48B	109.3
C23—C24—C19	120.5 (4)	H48A—C48—H48B	108.0
C23—C24—H24	119.8	N5—C49—C48	112.5 (3)
C19—C24—H24	119.8	N5—C49—C50	108.6 (3)
C22—C25—H25A	109.5	C48—C49—C50	112.5 (3)
C22—C25—H25B	109.5	N5—C49—H49	107.7
H25A—C25—H25B	109.5	C48—C49—H49	107.7
C22—C25—H25C	109.5	C50—C49—H49	107.7
H25A—C25—H25C	109.5	C43—C50—C49	112.2 (3)
H25B—C25—H25C	109.5	C43—C50—H50A	109.2
N4—C26—C33	111.5 (3)	C49—C50—H50A	109.2
N4—C26—C27	110.5 (3)	C43—C50—H50B	109.2
C33—C26—C27	109.6 (3)	C49—C50—H50B	109.2
N4—C26—H26	108.4	H50A—C50—H50B	107.9
C33—C26—H26	108.4	N5—C51—H51A	109.5
C27—C26—H26	108.4	N5—C51—H51B	109.5
C26—C27—C28	112.4 (3)	H51A—C51—H51B	109.5
C26—C27—H27A	109.1	N5—C51—H51C	109.5
C28—C27—H27A	109.1	H51A—C51—H51C	109.5
C26—C27—H27B	109.1	H51B—C51—H51C	109.5
C28—C27—H27B	109.1		

### Hydrogen-bond geometry (Å, º)

**Table:**

*D*—H···*A*	*D*—H	H···*A*	*D*···*A*	*D*—H···*A*
N2—H2′···O1^i^	0.85 (2)	2.10 (2)	2.935 (5)	171 (4)
N4—H4′···O2^ii^	0.86 (2)	2.05 (2)	2.894 (5)	166 (4)
N6—H6′···O3^iii^	0.86 (2)	2.30 (2)	3.164 (5)	174 (4)

Symmetry codes: (i) *x*, −*y*, *z*+1/2; (ii) *x*, −*y*+1, *z*+1/2; (iii) *x*, −*y*+2, *z*−1/2.
